# Developing a framework for a novel multi-disciplinary, multi-agency intervention(s), to improve medication management in community-dwelling older people on complex medication regimens (MEMORABLE)––a realist synthesis

**DOI:** 10.1186/s13643-017-0528-1

**Published:** 2017-07-03

**Authors:** Ian Maidment, Andrew Booth, Judy Mullan, Jane McKeown, Sylvia Bailey, Geoffrey Wong

**Affiliations:** 1 School of Life and Health Sciences, Aston University, Aston Triangle, Birmingham, B4 7ET UK; 2 School of Health and Related Research, University of Sheffield, Regent Court, 30 Regent Street, Sheffield, S1 4DA UK; 3 University of Wollongong, Northfields Ave, Wollongong, New South Wales 2522 Australia; 4 School of Nursing and Midwifery, University of Sheffield, Barber House Annexe, 3a Clarkehouse Road, Sheffield, S10 2LA UK; 5 Kingshayes Road, Aldridge, Walsall, West Midlands WS9 8RU UK; 6 Nuffield Department of Primary Care Health Sciences, Radcliffe Observatory Quarter, University of Oxford, Woodstock Road, Oxford, OX2 6GG UK

**Keywords:** Medication management, Older people, Patient safety, Primary care health, Polypharmacy

## Abstract

**Background:**

Medication-related adverse events have been estimated to be responsible for 5700 deaths and cost the UK £750 million annually. This burden falls disproportionately on older people. Outcomes from interventions to optimise medication management are caused by multiple context-sensitive mechanisms. The MEdication Management in Older people: REalist Approaches BAsed on Literature and Evaluation (MEMORABLE) project uses realist synthesis to understand how, why, for whom and in what context interventions, to improve medication management in older people on complex medication regimes residing in the community, work.

**Method:**

This realist synthesis uses secondary data and primary data from interviews to develop the programme theory. A realist logic of analysis will synthesise data both within and across the two data sources to inform the design of a complex intervention(s) to help improve medication management in older people.

1. Literature review
  The review (using realist synthesis) contains five stages to develop an initial programme theory to understand why processes are more or less successful and under which situations: focussing of the research question; developing the initial programme theory; developing the search strategy; selection and appraisal based on relevance and rigour; and data analysis/synthesis to develop and refine the programme theory and context, intervention and mechanism configurations.
2. Realist interviews
  Realist interviews will explore and refine our understanding of the programme theory developed from the realist synthesis. Up to 30 older people and their informal carers (15 older people with multi-morbidity, 10 informal carers and 5 older people with dementia), and 20 care staff will be interviewed.
3. Developing framework for the intervention(s)
  Data from the realist synthesis and interviews will be used to refine the programme theory for the intervention(s) to identify: the mechanisms that need to be ‘triggered’, and the contexts related to these mechanisms. Intervention strategies that change the contexts so the mechanisms are triggered to produce desired outcomes will be developed. Feedback on these strategies will be obtained.

**Discussion:**

This realist synthesis aims to develop a framework (underpinned by our programme theory) for a novel multi-disciplinary, multi-agency intervention(s), to improve medication management in community-dwelling older people on complex medication regimens.

**Systematic review registration:**

PROSPERO CRD42016043506

**Electronic supplementary material:**

The online version of this article (doi:10.1186/s13643-017-0528-1) contains supplementary material, which is available to authorized users.

## Background

Between 2010 and 2050, the number of older people living in the UK will nearly double from around 10 to 19 million [1]. Half of people aged 75 or more are living with two or more long-term conditions [2]. The Francis report emphasised that patients should be protected from avoidable harm [3]. Yet, medication-related adverse events have been estimated to be responsible for 5700 deaths, at a cost of £750 million to the UK health service, every year [4]. Furthermore, between 5 and 8% of unplanned hospital admissions in the UK are related to medication issues [5].

Safe and effective medication management is particularly challenging in older people with chronic or long-term conditions on multiple medications with some degree of frailty and/or cognitive impairment [6–12]. Older people, particularly those with dementia, may be more susceptible to medication-related adverse events and less able to identify when a medication error has taken place [6, 8, 13, 14].

Complex care pathways delivered by a diverse range of care staff are needed to support medication management [2, 15]. For instance, older people often attend multiple clinics and interact with multiple health and social care professionals including GPs, community pharmacists, district nurses, and various secondary care clinicians, all of whom may help to optimise their medication management [6, 15]. In addition, for many older people, formal and informal carers have a key and pivotal role and provide front-line support for their medication management needs [16, 17]. However, formal carers frequently lack the appropriate training to deliver this role and informal carers, who often have self-care difficulties themselves, struggle with the responsibility and find the role stressful and burdensome [17–21]. Recent NICE guidance has identified the need for a collaborative approach to supporting older people with long-term conditions in managing their medication(s) [22].

Realist approaches aim to understand how a given context affects any mechanism, to generate either a positive or a negative outcome [23, 24]. In other words, they explore the relationship between context, mechanism and outcome (CMO) configurations or CMOCs [23]. Our MEdication Management in Older people: REalist Approaches BAsed on Literature and Evaluation (MEMORABLE) study uses a realist synthesis to address the three steps within the Medical Research Council framework for Developing Complex Interventions: identifying the evidence base, identifying/developing theory and modelling process and outcomes [25]. An additional file shows an overview of the study in a flow chart (see Additional file 1). Recognised guidelines for ensuring the quality of the review and how it is reported (the RAMESES guidelines) will be followed [23]. In this study, the realist programme theories will be developed through a combination of secondary (literature review, in the form of a realist synthesis: work package 1) and primary (realist interviews: work package 2) data collection methods. In work package (WP) 3, we will use data from within and across WP1 and WP2 to further develop and refine the realist programme theory. The data collected from each work package will be continually analysed to enable emerging findings from one package to inform the other. Finally, the resulting refined realist programme theory will be used to design the framework for the intervention(s) (WP3).

## Methods/design

### Aim

The project aims to use realist synthesis including primary data collection to develop a framework for a novel multi-disciplinary, multi-agency intervention(s), to improve medication management in community-dwelling older people on complex medication regimens.

### Objectives

1. To understand how and why any potentially relevant interventions, to optimise medication management, work (or do not work) for particular groups of older people in certain circumstances.
2. To synthesize the findings from objective 1 into a realist programme theory of an intervention(s) to support older people living in the community manage their medication.
3. To use realist programme theory developed from objective 2 to inform the development of an intervention(s) to assist older people living in the community to manage their medication.

### WP1: literature review

#### Method

This literature review (in the form of a realist synthesis) comprises five stages:

1. *Focussing the synthesis*: the overall focus is to develop a realist programme theory for an intervention to support older people living in the community to manage their medication. The realist synthesis will be focussed on the most relevant strategies that would be needed to be used in the medicine management intervention developed.
2. *Developing initial programme theory:* a programme theory sets out how and why outcomes occur within an intervention [23, 26].
  We will iteratively consult with our stakeholder group, and search the literature to locate current theories [27]. This searching is more exploratory than the formal searching in step 3 and aims to rapidly identify a range of possibly relevant explanatory theories. Iterative discussions in the project team will aim to understand and synthesise the various theories into an initial coherent programme theory. The stakeholder group (that includes patient and public involvement) will provide content expertise to help synthesise the initial programme theory and later refinement.
  The initial programme theory (candidate theory) will be refined as the synthesis progresses with input from both the project team and stakeholder group. The theory will be mapped out as a series of outcome steps required for the final desired outcome, identifying intermediate outcomes that take place either sequentially or in parallel [28]. For each step (where possible at this stage), the relevant and associated context and mechanism for each outcome will be developed from data identified from our exploratory searches [28, 29].
3. *Developing a search strategy:* we will use a CIMO (Context, Intervention, Mechanisms, Outcome) question framework [30] to construct an initial sampling frame for medication management in older people. The initial CIMO framework is:
**C**ontext: older people living in the community, medication complexity, ethnicity. Subgroup: people with dementia living in the community.
**I**ntervention: based on previous work and literature reviews, possible interventions include support from formal or informal carers, education, medication review, self-management and tools (including technology) to support adherence.
**M**echanisms: the mechanism(s) triggered by the intervention will be identified by the programme theory.
**O**utcomes: quality of life, adherence, adverse events, carer burden and economic (care costs including residential care and hospitalisations).
  We will then select distinct subsets of literature from within the sampling frame with which to test emerging theory [29, 31]. At this point, we anticipate that we will be able to use a comprehensive sampling approach. However, if the size of the literature proves unmanageable, then we will employ a variety of appropriate sampling strategies (e.g. theoretical sampling, maximum variation sampling, extreme case sampling) to optimise the analytical value of the realist synthesis component, as specified by the methodology [32].
  The initial sampling frame will be used as the starting point for selection of ‘index papers’ from which suggested conceptual or contextual explanations will be identified, developed and explored by following links out to wider bodies of relevant literature [29, 31]. When retrieved data suggests certain mechanisms may be particularly important, the search techniques will be refined to identify data from other clinical environments where these mechanisms may also be in operation, so that we can better understand their behaviour under different contexts.
  Realist synthesis uses iterative, purposive sampling from a wide range of evidence to develop, refine, confirm and refute theories about how and why an intervention works, for whom, and in what circumstances [32]. Consequently, the search strategy will be developed iteratively and re-visited at predetermined milestones, using different permutations and additional concepts [29, 31]. Searching will be guided by the need to find data to develop the programme theory; and hence, additional searching may be needed.
  We will subsequently use ‘cluster searching’ to identify ‘clusters’ of data from related publications. This approach will add to the conceptual richness and contextual thickness of studies initially identified within the sampling frame constructed through conventional topic-based searching [31]. We will identify ‘sibling’ (i.e. directly linked outputs from a single study) and ‘kinship’ (i.e. associated papers with a shared contextual or conceptual pedigree) papers and reports to add richness of data while preserving both rigour and relevance [31]. Active pursuit of citation networks, using Google Scholar and Web of Science will be used to link index papers to the wider literature. Searching will continue until sufficient data is found (‘theoretical saturation’) to conclude that a candidate programme theory is sufficiently coherent and plausible [29].
  We will use the most up-to-date methodological literature when devising search strategies relating to older people or medication management [33]. Based on previous work, international guidance and discussions with information specialists, sources will include Scopus, Web of Science, EMBASE/PubMed/MEDLINE, Cochrane, CINAHL, PsycINFO, ProQuest, Sociological Abstracts, Google Scholar, BASE (Bielefield Academic Search Engine)/ETHOS (British Library Electronic Thesis Online)/ProQuest Dissertations and Thesis, Grey Literature in Europe (http://www.opengrey.eu/) and NHS Evidence and equivalent, external experts and charities/user groups, and reference lists of relevant papers [29, 34].
4. *Selection and appraisal:* inclusion and exclusion are based on [29, 35]:
  Does the document contain any data that can contribute to developing or testing theory (*relevance*)?
  Are the methods (if any) utilised to generate the relevant data trustworthy and credible (*rigour*)?
  Selection and appraisal is a two-step process:
  1. Potentially relevant documents will initially be screened by title, abstract and key words [29, 31] by the Research Associate (RA). A 10% random sample will be checked by AB and GW for consistency (any disagreements will be resolved with the input of IM).
  2. The full texts of this set of documents will be obtained and screened by the RA. Again, a 10% random sample will be checked by AB and GW for consistency using the same approach as outlined above.
  The full texts of all relevant documents will be imported into NVivo (a qualitative data analysis software tool). Relevant data from included documents will be coded into NVivo. Some of the codes will come from the programme theory (i.e. deductive coding). Others will come from the data (i.e. inductive coding). These codes will cover concepts that are judged to be important and potentially relevant to the programme theory. When coding, where it is possible to make such inferences, data will be coded as context, mechanism or outcome. Any data that informs the relationship of data within Context-Mechanism-Outcome-Configurations (CMOCs) or between the CMOCs (contained within the programme theory) will also be coded [23, 35].
5. *Data analysis and synthesis:* This will configure the coded data to develop the CMOCs within the programme theory by piecing together data from different sources [23]. Relevant data will be interpreted as being about context, mechanism and/or outcome within the CMOCs contained within our overarching programme theory. These data may come from a range of included documents or from the interview transcripts (see WP2 below). The configurations will be presented and discussed and debated within our regular project team meetings. We will ask a number of questions about the data informed by the approach used in Wong et al. (see Box 1 in the reference [27]). These questions will cover relevance, interpretation of meaning, judgement regarding the CMOCs, judgements about the programme theory and rigour. Part of the process of data analysis will involve making inferences about the relationships of the CMOCs to the programme theory [23]. Where needed, we will attempt to link our findings to substantive theories to further refine our theoretical understanding; for example, social cognitive theory is frequently referenced in connection with medication self-management and may help to explain some aspects of the programme theory [36, 37].

### WP2: realist interviews

Realist interviews are a type of qualitative interview where the researcher does a ‘show and tell’ with the participant [38]. Realist studies are not constructivist [39]. Therefore, unlike other qualitative interviewing, in realist interviews, the interviewer starts with some ideas about (for example) how and why an intervention might ‘work’––informed by their programme theory. They then direct the interviews in such a way as to ‘test’ the hypothesis contained within their programme theory [39]. The participant is initially asked a series of general questions about the topic area (i.e. is ‘eased in’) and then questioned in the most neutral way possible about aspects of the programme theory. An additional file shows an outline interview schedule (see Additional file 2). Realist interviews will gather additional data to confirm, refute or refine aspects of the programme theory developed from the literature review work package (WP1) [40]. The intention is that these interviews will be used to further develop and refine aspects of the programme theory that remain unclear based on the analyses of data from the realist synthesis. Or they may surface potentially relevant data about aspects of the programme theory that have not been found in the literature.

Method: Ethical approvals for these interviews including any with vulnerable populations will be sought prior to the start of this WP. Focus groups and/or interviews with older people, informal carers and care staff will be recorded and transcribed verbatim. We will ascertain from the participants whether focus groups or one-to-one interviews are most appropriate. We are mindful of the sensitive issues which may be discussed, and some people may prefer a one-to-one interview although some may find the group environment empowering. Based on our practical experience, one-to-one interviews are likely to be needed for care staff.

Sample size: Up to 30 older people and their informal carers (15 older people with multi-morbidity, 10 informal carers and 5 older people with dementia) and 20 health and social care staff (including formal carers). Previous experience indicates that this will generate sufficient data for an in-depth analysis from multiple perspectives [41, 42]. However, interviews will stop when theoretical saturation is reached. The sampling strategy will be informed by the data collected from WP1 and be directed by the need to find data relevant to explore and refine aspects of the programme theory developed in WP1. Sampling of participants will be as follows:

- Interviews with older people and informal carers: Participants will be purposively sampled to ensure diversity in potentially conceptually relevant characteristics (for example) including age, ethnicity, gender, number of co-morbidities, presence of dementia and level of support from carer.
- One-to-one interviews with health and social care professionals (including GPs, social workers, formal carers, nurses, community pharmacists, secondary care consultants) who support older people in medication management. Participants will be purposively sampled to ensure diversity in potentially conceptually relevant characteristics (for example) including: locality (rural vs. urban) and the index of deprivation in the area that they work.

Data analysis: NVivo software will be used to organise the qualitative data. The process of coding the data from the transcripts of the interviews/focus groups will be similar to that outlined above in WP1.

Data coding and analysis will initially be conducted by the RA. One member of the team (GW) will independently check 10% of interviews for consistency in coding. Data analysis will take place after each interview/focus group and use a realist logic of analysis. Through discussion and disputation, the project team will make inferences with respect to how the programme theory should be further refined (or not) based on the additional data from the realist interviews. In other words, asking the question how and why do these findings inform (if at all) the programme theory from WP1 and what refinements (if any) do we need to make to it? As quality control processes, (a) transcripts will be shared with participants and feedback elicited as to their veracity, and (b) a 10% sample of transcripts will be checked for consistencies in coding, interpretations and inferences made by GW. Any disagreements will be resolved by discussion between the RA, GW and IM, with IM being the final arbiter.

### WP3: developing the framework for an intervention(s)

The data from the realist synthesis (literature review) and realist interviews will be used to develop a framework for an intervention(s) to support community-dwelling older people, including people with dementia, manage their medication. The framework will be underpinned and informed by our programme theory and is essentially the result of those programme theories that have been prioritised through the literature searching and stakeholder consultation and then brought together to form a coherent explanation for intervention effectiveness. When designing the intervention, we may need to also use other types of knowledge that is not explicitly articulated within the programme theory. For example, our programme theory may inform us about which intervention strategies, we need to use within the intervention, but not provide enough detail for us to be able to stipulate who is ideally placed to deliver the strategy or how often. When we implement the intervention, we may thus also need to draw on other knowledge (such as from stakeholders) to be able to ‘fill in’ such a gap.

If needed, at this stage, we will undertake additional searching and/or interviews to find additional relevant data to refine the programme theory. However, we anticipate that by this stage, we will have found enough data for us to reach theoretical saturation. We will be able to combine the data from these two sources as we will be using the same logic of analysis for both the interview data and that from the realist synthesis [32].

To move from programme theory to intervention(s) design, we will:

- Use the data from the realist synthesis and realist interviews to identify the most important mechanisms within the programme theory that need to be ‘triggered’ to get desired outcomes
- Identify which contexts are related to these ‘key’ mechanisms (i.e. which CMOCs are the mechanisms found in)
- Draw on data from the realist synthesis that provides information of the intervention strategies that can change the contexts in the relevant ‘key’ CMOCs. In other words, for this last stage, we will seek information on which intervention strategies we might be able to use to change the contexts in such a way that ‘key’ mechanisms are triggered to produce desired outcomes [23]. Ultimately, the analysis will provide information on the required intervention strategies [28, 40, 43].

These strategies will be presented to a project event involving 30 care staff plus 10 PPI reps to obtain detailed feedback on our proposed intervention strategies. This will include discussion on the plausibility, feasibility and relevance to patients and the NHS. The data from this event will be analysed and the outputs presented to the stakeholder group providing information on the required intervention strategies and thus the framework to formulate an intervention(s) for future feasibility testing.

## Discussion

Medication management in older people on polypharmacy is one of the key challenges in modern healthcare. This project aims to develop a framework for novel multi-disciplinary, multi-agency intervention(s), to improve medication management in community-dwelling older people on complex medication regimens. An additional file reports the study against PRISMA-P criteria (see Additional file 3). Realist approaches aim to answer the question; “What works for whom, in what circumstances, how and why?” We have chosen this approach because it is ideally suited to explore how and why a complex social programme involving human actions and decisions, such as medication management, may or may not work, and thus to inform the theoretical development of an intervention(s). As far as we are aware, this is one of the first studies to utilise realism in medication management. It is also one of the first projects to deliberately combine a realist synthesis with primary data collected from realist interviews to ensure that sufficient relevant data is found for programme theory development and ‘testing’. As such, it is methodologically novel and adds to the literature on the use of realist-based approaches to inform the development of programmes [44] and offers an alternative to the use of the Theoretical Domains Framework to explore mediators for behavioural change [45, 46].

We have anticipated a challenge common to many realist syntheses, namely, that of finding enough data for programme theory development and testing. To address this issue, we have deliberately built in the following two strategies in our project––we have expert and novel searching expertise in our project team (AB) and will use realist interviews to gather additional relevant data.

Our main goal is to develop the framework (underpinned and informed by our programme theory) for an intervention(s), to improve medication management in community-dwelling older people on complex medication regimens. However, in pursuing this goal, we will use our interpretative research approaches to make sense of the issues and challenges in this area. We will identify what is likely to be effective and conversely, what is not likely to be effective, in what contexts and for whom. We believe that our increased understanding will already yield value to the audiences outlined below, in advance of moving on to piloting the candidate intervention, as follows:

1. Outputs for policy, decision makers and clinicians: our findings may have implications for how current medication optimisation programmes should be delivered for community-dwelling older people.
2. Outputs for service users and third sector organisations: we will develop information on how to make better use of existing medication management encounters.
3. Outputs for academics/researchers: the findings from our research are likely to have relevance to other national and international researchers planning to develop similar interventions.

## Additional files

Additional file 1: Study flow chart. (DOC 68 kb)

Additional file 2: Interview schedule for realist interviews in WP2. (DOCX 12 kb)

Additional file 3: Checklist against PRISMA-P criteria. (DOCX 29 kb)

## Abbreviations

CIMO: Context, Intervention, Mechanisms, Outcome
CMO: Context, Mechanism, Outcome
CMOCs: Context, Mechanism, Outcome configurations
MEMORABLE: MEdication Management in Older people: REalist Approaches BAsed on Literature and Evaluation
NICE: National Institute for Health and Care Excellence
PPI: Patient Public Involvement
PRISMA-P: Preferred Reporting Items for Systematic Review and Meta-Analysis Protocols
RAMESES: Realist and Meta-narrative Evidence Synthesis
WP: Work package
