# Supplementary material for: Developing a framework for a novel multi-disciplinary, multi-agency intervention(s), to improve medication management in community-dwelling older people on complex medication regimens (MEMORABLE)––a realist synthesis
Source: Syst Rev. 2017 Jul 3;6:125. doi: 10.1186/s13643-017-0528-1 (PMC5496371; doi:10.1186/s13643-017-0528-1)
Supplement: Supplementary file 1 — Study flow chart. (DOC 68 kb) [file 13643_2017_528_MOESM1_ESM.doc]

**Flow diagram**

**Flow Chart: Developing a framework for a novel multi-disciplinary, multi-agency intervention(s), to improve medication management in community-dwelling older people on complex medication regimens**

**Project Start Up:**

- Establish Project Team

- Establish Stakeholder Group

**Work Package 1: Realist Review**

- Focussing synthesis

- Developing initial Programme Theory

- Developing Search Strategy

- Article Selection (relevance/rigour)

- Data analysis and synthesis

**Work Package 2: Realist Interviews**

- Realist Interviews (up to 30 older people/carers/people with dementia and 20 Care Professionals)

- Data analysis

**Work Package 3: Developing Framework for Intervention(s) and Dissemination (Integration of WP1 and WP2)**

- Refine Programme Theory– additional interviews/searching as required

- Identify key mechanisms and related contexts

- Identify and develop intervention strategies needed to change context and trigger mechanism

- Framework for intervention(s) presented to Project Event and further refined.

- Input sought from Stakeholder Group

- Dissemination

- An understanding of what works for whom and what circumstances.

- Rigorously theorised intervention(s) to improve medication management in older people in complex medication in the community

- Outputs for other researchers, policy makers, clinicians, older people, carers, third sector organisations
