# Supplementary material for: Developing a framework for a novel multi-disciplinary, multi-agency intervention(s), to improve medication management in community-dwelling older people on complex medication regimens (MEMORABLE)––a realist synthesis
Source: Syst Rev. 2017 Jul 3;6:125. doi: 10.1186/s13643-017-0528-1 (PMC5496371; doi:10.1186/s13643-017-0528-1)
Supplement: Supplementary file 2 — Interview schedule for realist interviews in WP2. (DOCX 12 kb) [file 13643_2017_528_MOESM2_ESM.docx]

**Appendix 2: Outline Interview Schedule**

Provisionally the interviews and focus groups will explore the questions listed below. However, refinement of these questions might need to occur, based on the findings of the programme theory developed in Work Package 1. Within a realist interview, participants are asked to provide their interpretations and perceptions of aspects of the programme theory. Care however must be taken to set the questions up in such a way that social desirability responding is avoided. As such, the questioning starts with an unfocussed or a more general discussion about the topic area and then gradually ‘drills’ down into different sections of the programme theory.

Examples of ‘opening’ questions for patients: Tell me about the different illnesses you have? Do you take any medications for your illnesses (which ones)? Tell me about your experience of taking these medicines? Does anyone help you to take them? Who? And how do they help you? Can you think of anything that might make it easier to help you take your medication? How and why would that help you?

Examples of questions that link to programme theory for patients: When I have spoken to other people, they have told me that X, Y and Z have helped them as well. What do you think? Why? When do you think X, Y and Z would help you? Why? Some people have also told me that A, B and C gets in the way of taking their medication. What do you think? Why?

Examples of ‘opening’ questions for care professionals: Are there times when you help patients with their medication? Can you tell me about how you help them? Why do you need to?

Examples of questions that link to programme theory for care professionals: When I have spoken to health care professionals, they have told me that X, Y and Z have helped patients to manage their medication better. What do you think? Why? When do you think X, Y and Z would help a patient? Why? Are there certain patients that X, Y and Z might help more? Or when X, Y and Z might not help? Why? Some health care professionals have also told me that A, B and C gets in the way of patients taking their medication as prescribed. What do you think? Why?
